# Identification of potential autophagy-related genes in steroid-induced osteonecrosis of the femoral head via bioinformatics analysis and experimental verification

**DOI:** 10.1186/s13018-022-02977-x

**Published:** 2022-02-12

**Authors:** Xue-Zhen Liang, Di Luo, Yan-Rong Chen, Jia-Cheng Li, Bo-Zhao Yan, Yan-Bo Guo, Ming-Tao Wen, Bo Xu, Gang Li

**Affiliations:** 1 Orthopaedic Microsurgery, Affiliated Hospital of Shandong University of Traditional Chinese Medicine, 16369 Jingshi Road, Jinan City, 250014 Shandong Province China; 2 The First Clinical Medical School, Shandong University of Traditional Chinese Medicine, Shandong, 250355 China

**Keywords:** Steroid-induced osteonecrosis of the femoral head, Autophagy, Bioinformatics analysis, Gene Expression Omnibus

## Abstract

**Purpose:**

Steroid-induced osteonecrosis of the femoral head (SONFH) is a refractory orthopaedic hip joint disease that occurs in young- and middle-aged people. Previous experimental studies have shown that autophagy might be involved in the pathological process of SONFH, but the pathogenesis of autophagy in SONFH remains unclear. We aimed to identify and validate the key potential autophagy-related genes involved in SONFH to further illustrate the mechanism of autophagy in SONFH through bioinformatics analysis.

**Methods:**

The GSE123568 mRNA expression profile dataset, including 10 non-SONFH (following steroid administration) samples and 30 SONFH samples, was downloaded from the Gene Expression Omnibus (GEO) database. Autophagy-related genes were obtained from the Human Autophagy Database (HADb). The autophagy-related genes involved in SONFH were screened by intersecting the GSE123568 dataset with the set of autophagy genes. The differentially expressed autophagy-related genes involved in SONFH were identified with R software. In addition, Gene Ontology (GO) and Kyoto Encyclopedia of Genes and Genomes (KEGG) pathway enrichment analyses of the differentially expressed autophagy-related genes involved in SONFH were conducted by using R software. Then, the correlations between the expression levels of the differentially expressed autophagy-related genes involved in SONFH were confirmed with R software. Moreover, the protein–protein interaction (PPI) network was analysed by using the Search Tool for the Retrieval of Interacting Genes (STRING), significant gene cluster modules were identified with the MCODE Cytoscape plugin, and hub genes among the differentially expressed autophagy-related genes involved in SONFH were screened by using the CytoHubba Cytoscape plugin. Finally, the expression levels of the hub genes of the differentially expressed autophagy-related genes involved in SONFH were validated in hip articular cartilage specimens from necrotic femur heads (NFHs) by using the GSE74089 dataset and further verification by qRT-PCR.

**Results:**

A total of 34 differentially expressed autophagy-related genes were identified between the peripheral blood samples of SONFH patients and non-SONFH patients based on the defined criteria, including 25 upregulated genes and 9 downregulated genes. The GO and KEGG pathway enrichment analyses revealed that these 34 differentially expressed autophagy-related genes involved in SONFH were particularly enriched in death domain receptors, the FOXO signalling pathway and apoptosis. Correlation analysis revealed significant correlations among the 34 differentially expressed autophagy-related genes involved in SONFH. The PPI results demonstrated that the 34 differentially expressed autophagy-related genes interacted with each other. Ten hub genes were identified by using the MCC algorithms of CytoHubba. The GSE74089 dataset showed that TNFSF10, PTEN and CFLAR were significantly upregulated while BCL2L1 was significantly downregulated in the hip cartilage specimens, which was consistent with the GSE123568 dataset. TNFSF10, PTEN and BCL2L1 were detected with consistent expression by qRT-PCR.

**Conclusions:**

Thirty-four potential autophagy-related genes involved in SONFH were identified via bioinformatics analysis. TNFSF10, PTEN and BCL2L1 might serve as potential drug targets and biomarkers because they regulate autophagy. These results expand the autophagy-related understanding of SONFH and might be useful in the diagnosis and prognosis of SONFH.

## Introduction

Steroid-induced osteonecrosis of the femoral head (SONFH) is a chronic refractory orthopaedic disease characterized by accumulation of microfractures without continuous remodelling or the collapse of the femoral head, with high mortality, disability and dysfunction [1, 2]. SONFH occurs most frequently in young- and middle-aged adults, and without effective systematic treatment, approximately 80% of SONFH patients develop femoral head collapse within 1 ~ 4 years and then require artificial joint replacement [3]. Although glucocorticoids (GCs) have been recognized as a risk factor for SONFH, their exact pathogenesis has not been well defined. As the SONFH study deepened, accumulating highly recognized mechanistic hypotheses of pathogenicity have been proposed to explain their developmental mechanisms, including uncoupled bone remodelling or unbalanced osteogenic and adipogenic differentiation of BMSCs, cell proliferation and programmed cell death [4]. Among these biological processes, programmed cell death plays a critical role in the development of SONFH. Autophagy, a programmed cell death-like process, has been identified after SONFH, but the mechanism of autophagy in bone repair and bone homeostasis remains unclear.

Autophagy is an evolutionarily conserved intracellular catabolic process that contributes to the degradation of accumulated and unnecessary intracellular materials in lysosomes, which differs from apoptosis [5]. The discovery of autophagy mechanisms was recognized by the 2016 Nobel Prize in Physiology or Medicine [6]. Recent studies have revealed that autophagy is related to various diseases including SONFH, and the expression levels of some key targets change after autophagy [7–10]. For instance, the proteins necessary for autophagy, Atg5, Atg7, Atg4B and LC3, play a key role in the production of osteoclast crinkle boundaries and the secretory function of osteoclasts and in vitro and in vivo in maintaining balance and homeostasis[11]. Moreover, FIP200 is an integral part of the ULK-Atg13-FIP200 complex and regulates autophagy in osteoblasts, which affects bone fragility and bone loss [12]. In addition, it has been reported that several signalling pathways influence the biological function of SONFH through autophagy. The mTOR pathway is the main regulator of mammalian autophagy, and oestradiol has recently been found to increase the level of autophagy through the ER-ERK-mTOR pathway to prevent osteoblast apoptosis in SONFH [13]. However, many autophagy-related genes involved in SONFH have not yet been found, so further studies of autophagy-related genes involved in SONFH genes need to be explored.

To date, there have been no bioinformatics-based studies on the pathogenesis of autophagy-related genes after SONFH. Zhang Y generated a SONFH-related dataset, GSE123568, based on the genome-wide analysis of human peripheral serum, which included both normal patient serum samples and SONFH patient serum samples [14]. Hence, we could use machine learning algorithms and multiple bioinformatic approaches to screen the differentially expressed genes between SONFH patients and normal individuals based on this microarray dataset. In this study, we intersected these differentially expressed genes with the ferroptosis dataset to obtain differentially expressed autophagy-related genes. Then, the Gene Ontology (GO) and Kyoto Encyclopedia of Genes and Genomes (KEGG) databases were used for the enrichment analysis of differentially expressed autophagy-related genes to establish the pathogenesis of SONFH. Moreover, correlation analysis was performed to explore the association of the identified differentially expressed autophagy-related genes with each other. Furthermore, we constructed a protein–protein interaction (PPI) network to identify hub genes. Finally, the expression levels of crucial differentially expressed autophagy-related genes were further verified in the GSE74089 dataset based on hip cartilage specimens. Therefore, our results reveal potential autophagy-related genes involved in SONFH, help to understand the pathogenesis of autophagy in SONFH and provide potential biomarkers for the clinical diagnosis and treatment of SONFH.

## Materials and methods

### Autophagy-related gene datasets and microarray data

The Human Autophagy Database (HADb) is a human autophagy-dedicated database. It is a public repository containing information about the human genes identified as being involved in autophagy to date (http://www.autophagy.lu/index.html) [15].

The GSE123568 mRNA expression profile dataset was obtained from the National Center for Biotechnology Information Gene Expression Omnibus (NCBI GEO, https://www.ncbi.nlm.nih.gov/geo/) database according to the following inclusion criteria: (1) “Steroid-induced Osteonecrosis of the Femoral Head” or “SONFH”; (2) *Homo sapiens*; and (3) “Expression Profiling by array” or “Expression profiling by high throughput sequencing”. GSE123568 was submitted on 10 Dec 2018 and updated on 01 Jan 2020 by Zhang et al. It includes 10 non-SONFH (following steroid administration) samples and 30 SONFH samples (https://www.ncbi.nlm.nih.gov/geo/query/acc.cgi?acc=GSE123568), and the platform on which the dataset was generated was the GPL15207 [PrimeView] Affymetrix Human Gene Expression Array.

### Differential expression analysis of autophagy-related genes in SONFH

To preprocess and normalize the original files of GSE123568, we downloaded the CEL files containing the raw data of GSE123568, and used the “affy” R package based on the robust multiarray average (RMA) method. To observe the overall distribution in non-SONFH samples and SONFH samples and identify the presence of singular samples, we projected the high-dimensional repeatability of data GSE123568 data onto two-dimensional space for principal component analysis (PCA). The expression values of these autophagy-related genes in the GSE123568 dataset were first identified and matched. To identify the differentially expressed autophagy-related genes in SONFH, we used the “limma” R package, and an adjusted P value < 0.05 and |log_2_ fold change (FC)|> log_2_1.5 were the criteria for screening differentially expressed genes. The PCA, heatmaps and volcano plots were computed and visualized by using the “PCA”, “pheatmap” and “ggplot2” R packages.

### Functional enrichment analysis of autophagy-related genes in SONFH

To analyse the identified differentially expressed autophagy-related genes involved in SONFH, GO and KEGG pathway enrichment analyses were conducted and visualized in R software using the “clusterProfiler” and “GOplot” R packages. The GO enrichment analysis was conducted according to the main categories biological process (BP), cellular component (CC), and molecular function (MF). An adjusted P value < 0.05 was considered the screening criterion for significantly enriched terms.

### Correlation analysis of the differentially expressed autophagy-related genes in SONFH

To confirm the correlation between the expression levels of differentially expressed autophagy-related genes in SONFH, we used the Spearman correlation coefficient in the “corrplot” R package.

### Construction of the PPI network and screening of hub genes and key modules

To gain more insight into the in-depth relationships of differentially expressed autophagy-related genes in SONFH, we analysed and constructed a PPI network using the Search Tool for Recurring Instances of Neighbouring Genes (STRING, Version: 11.0, https://string-db.org/) and Cytoscape software (version: 3.7.2, http://cyto-scape.org/). The thresholds for the combined score were set to a medium confidence > 0.4, and the isolated nodes were discarded. We used the molecular complex detection (MCODE) plugin for the clustering analysis of gene networks to select the key subnetwork modules. We explored important nodes/hubs and fragile motifs in an interactome network by using the CytoHubb plugin, which provided 11 topological analysis algorithms including the Degree, Edge Percolated Component (EPC), Maximum Neighbourhood Component (MNC), Density of Maximum Neighbourhood Component (DMNC), and Maximal Clique Centrality (MCC) algorithms, and centralities based on shortest paths, such as the Bottleneck (BN), EcCentricity, Closeness, Radiality, Betweenness and Stress paths.

### Cross-validation of the external dataset for the differentially expressed autophagy-related hub genes in SONFH

The GSE74089 mRNA expression dataset related to necrotic femur heads (NFHs), which included 4 hip cartilage samples from patients with NFHs and 4 hip cartilage samples from healthy controls, was downloaded from the GEO database. The platform used to generate the dataset was GPL13497 Agilent-026652 Whole Human Genome Microarray 4 × 44 K v2. To achieve cross-validation, we analysed the expression levels of differentially expressed autophagy-related hub genes in the GSE74089 dataset and then compared them with the expression levels in GSE123568 dataset in this study.

A total of 12 cartilage samples (including 6 SONFH and 6 healthy control samples) were collected from the Affiliated Hospital of Shandong University of Traditional Chinese Medicine. The Ethical Committee of the Affiliated Hospital of Shandong University of Traditional Chinese Medicine approved this study, and the respective patients provided written informed consent. During artificial hip replacement, femoral neck fracture and SONFH samples were collected, the cartilage (1 * 1 cm^2^) was immediately frozen in liquid nitrogen, and the RNA was extracted according to an established protocol. The mRNA transcripts were quantified by qRT-PCR using Thermo Scientific™ NanoDrop Lite and a CFX96 Touch Real-Time PCR Detection System (BIO-RAD, CFX96, USA).

The amplification conditions were set as follows: 95 °C for 10 min, followed by 40 cycles of 95 °C for 15 s, 60 °C for 30 s and 60 °C for 30 s. Then, a melting curve was established to obtain the experimental data. GAPDH was used as the reference gene, and all qRT-PCR reactions were tested in triplicate. A standard comparative method (ΔΔCt) was used to evaluate the expression stability of the potential candidate genes. Relative target gene expression levels were calculated using the 2^−ΔΔCt^ method. The sequences of primers used in this study are listed in Table 1.

**Table 1:** Primer sequences and respective amplicon lengths used for the qRT-PCR assays

Gene symbol	Forward primer (5'–3’)	Reverse primer (5'–3’)	Amplicon length (bp)	Annealing Tm° C
GAPDH	GTGAGATCGGTAGGTTGGTG	CCTTGACTTTGAGCTCGTGA	159	60
TNFSF10	GTCAAGTGGCAACTCCGTCAG	TGTGTTGCTTCTTCCTCTGGTC	168	60
PTEN	TATTCCCAGTCAGAGGCGCTAT	ACAGGTAACGGCTGAGGGAAC	246	60
CFLAR	TGTCGGGGACTTGGCTGAAC	AGTCCGAAACAAGGTGAGGGT	125	60
BCL2L1	GTGGAACTCTATGGGAACAATGC	AAGAGTGAGCCCAGCAGAAC	116	60

### Statistical analysis

Statistical analysis of the data was performed using R software (version 4.0.2) in this study. The gene expression levels of the GSE123568 and GSE74089 samples were compared using the Kruskal–Wallis test. The gene expression levels of samples in the qRT-PCR samples were compared by independent samples Student’s t-test or the Mann–Whitney U test as appropriate. *p* < 0.05 was set as the threshold for significant statistical significance.

## Results

### Differentially expressed autophagy-related genes in the retrospective SONFH- related analysis of autophagy-related genes

**Fig. 1:**
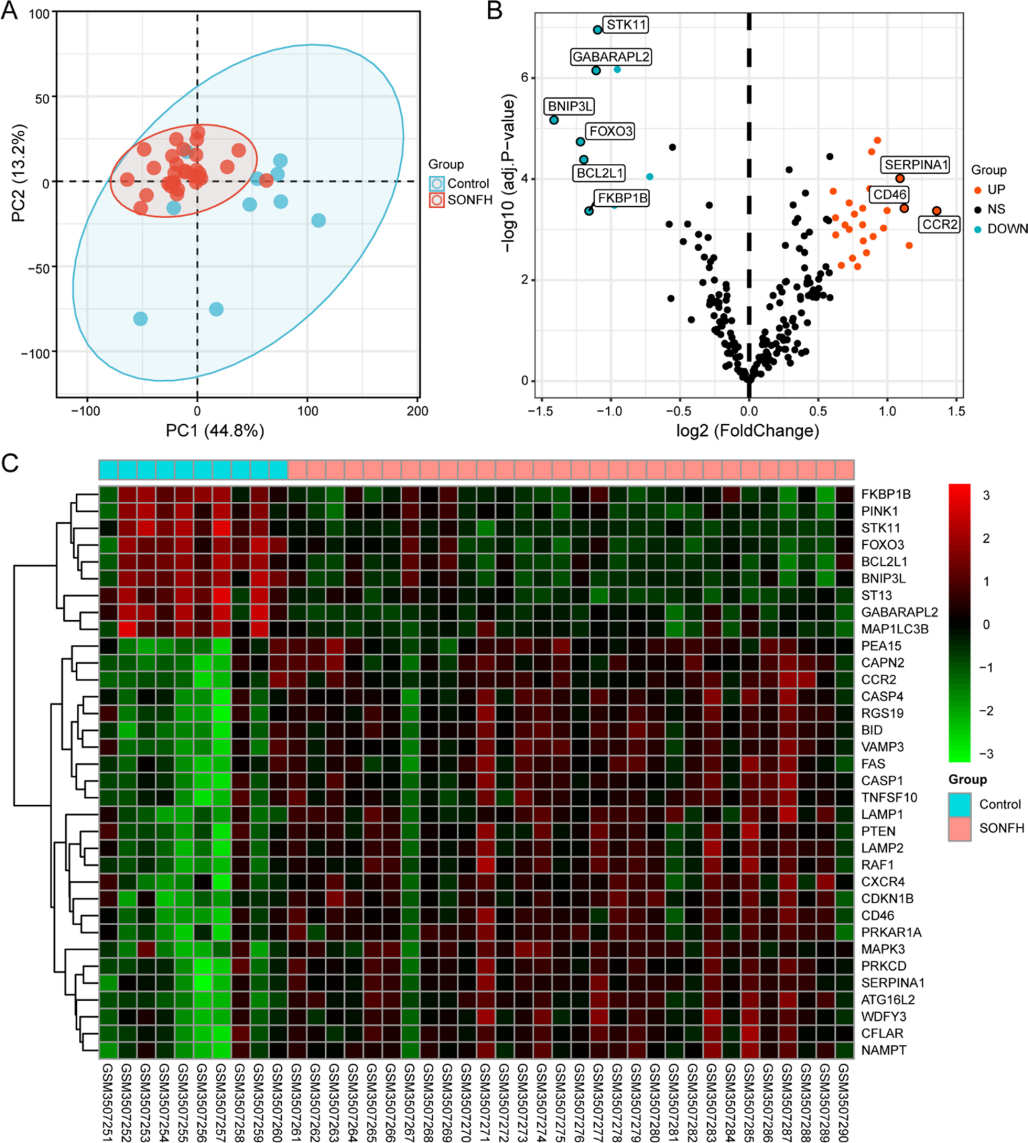
Differentially expressed autophagy-related genes in SONFH and non-SONFH samples. **a** Principal component analysis of GSE123568. **b** Volcano plot of 222 differentially expressed autophagy-related genes. The red dots represent the significantly upregulated genes and the blue dots indicate the significantly downregulated genes. **c** Hierarchical clustering heatmap of the 34 differentially expressed autophagy-related genes in SONFH and non-SONFH samples

To confirm the cluster analysis data and evaluate the repeatability of GSE123568, we subjected the data to PCA, and the results showed that the repeatability of the data in the GSE123568 dataset was reliable, as indicated in Fig. 1a. A total of 222 nonduplicated genes were excavated from HADb, which were then compared with the GSE123568 dataset to reveal 34 differentially expressed autophagy-related genes based on the defined criteria of an adjusted P value < 0.05 and |logFC|> log_2_1.5, including 25 upregulated genes and 9 downregulated genes in SONFH samples relative to non-SONFH samples.

**Fig. 2:**
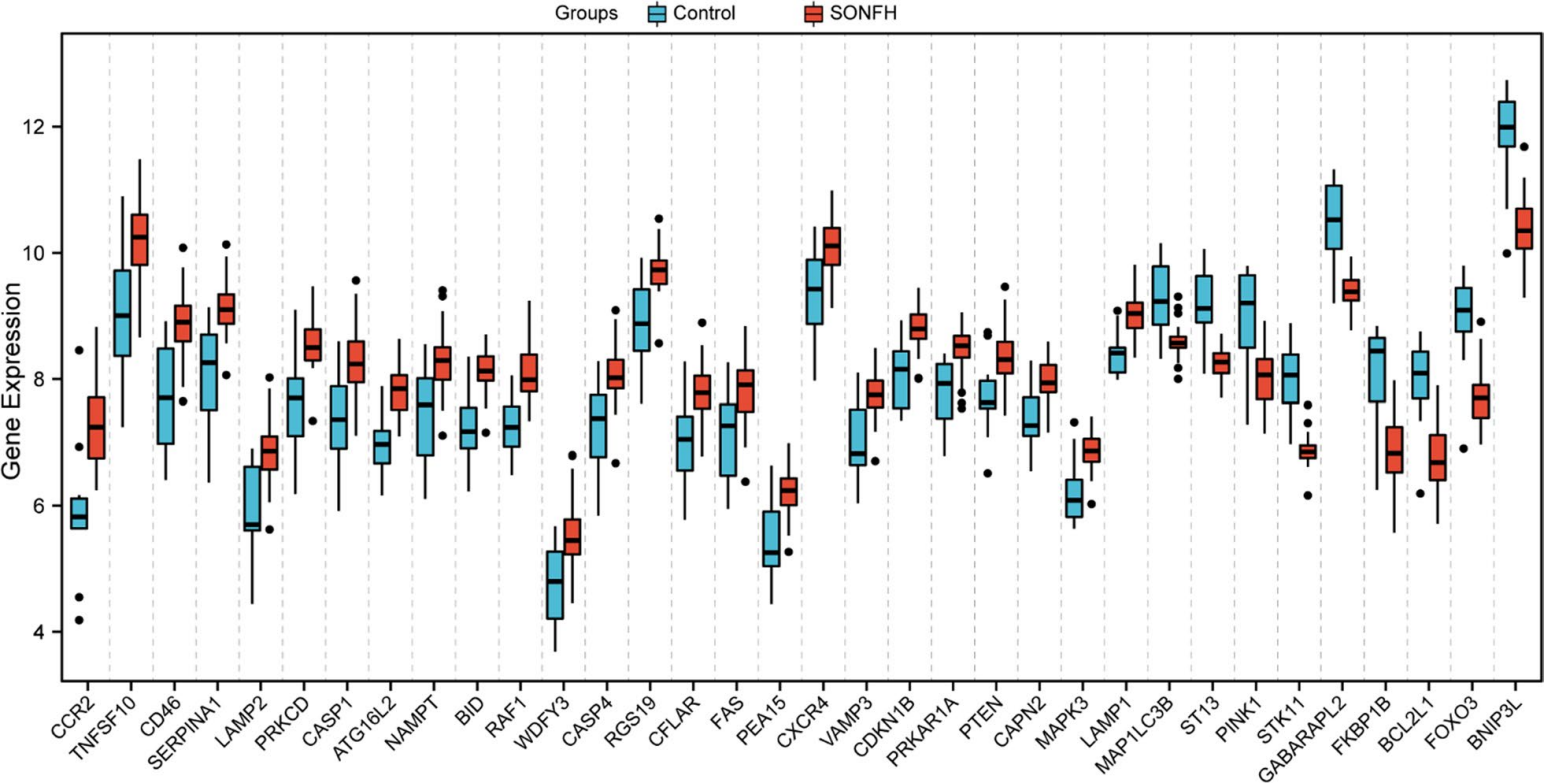
Boxplot of the 34 differentially expressed autophagy-related genes in SONFH and non-SONFH samples

The 34 differentially expressed autophagy-related genes of GSE123568 are shown in Fig. 1b, c in volcano plots and a hierarchical clustering heatmap generated by the “ggplot” and “pheatmap” R packages, respectively. The expression patterns of the 34 differentially expressed autophagy-related genes identified between the SONFH and non-SONFH samples are shown in the box plot in Fig. 2 and listed in Table 2.

**Table 2:** 34 differentially expressed autophagy-related genes in SONFH samples relative to non-SONFH samples

Gene ID	Name	Symbol	logFC	P value	Adj. P. val
729,230	Chemokine (C–C motif) receptor 2	CCR2	1.357	2.18E−05	0.000425
8743	Tumour necrosis factor (ligand) superfamily, member 10	TNFSF10	1.157	0.000195	0.002057
4179	CD46 molecule, complement regulatory protein	CD46	1.122	1.84E−05	0.000378
5265	Serpin peptidase inhibitor, clade A (alpha-1 antiproteinase, antitrypsin), member 1	SERPINA1	1.091	2.83E−06	9.69E−05
3920	Lysosomal-associated membrane protein 2	LAMP2	0.998	2.15E−05	0.00042
5580	Protein kinase C, delta	PRKCD	0.988	6.25E−06	0.000173
834	Caspase 1, apoptosis-related cysteine peptidase (interleukin 1, beta, convertase)	CASP1	0.972	6.74E−05	0.000934
89,849	ATG16 autophagy-related 16-like 2 (S. cerevisiae)	ATG16L2	0.928	2.4E−07	1.71E−05
10,135	Nicotinamide phosphoribosyltransferase	NAMPT	0.897	0.000113	0.001367
637	BH3 interacting domain death agonist	BID	0.886	4.81E−07	2.88E−05
5894	v-raf-1 murine leukaemia viral oncogene homolog 1	RAF1	0.872	5.37E−06	0.000155
23,001	WD repeat and FYVE domain containing 3	WDFY3	0.848	0.000309	0.002868
837	Caspase 4, apoptosis-related cysteine peptidase	CASP4	0.825	0.000148	0.001672
10,287	Regulator of G-protein signalling 19	RGS19	0.821	1.83E−05	0.000377
8837	CASP8 and FADD-like apoptosis regulator	CFLAR	0.821	5.42E−05	0.000803
355	Fas (TNF receptor superfamily, member 6)	FAS	0.784	0.000757	0.005384
8682	Phosphoprotein enriched in astrocytes 15	PEA15	0.761	2.75E−05	0.000493
7852	Chemokine (C-X-C motif) receptor 4	CXCR4	0.747	0.000432	0.003674
9341	Vesicle-associated membrane protein 3 (cellubrevin)	VAMP3	0.724	7.26E−05	0.000989
1027	Cyclin-dependent kinase inhibitor 1B (p27, Kip1)	CDKN1B	0.723	1.32E−05	0.000296
5573	Protein kinase, cAMP-dependent, regulatory, type I, alpha (tissue specific extinguisher 1)	PRKAR1A	0.693	5.49E−05	0.000809
5728	Phosphatase and tensin homolog	PTEN	0.666	0.000695	0.0051
824	Calpain 2, (m/II) large subunit	CAPN2	0.626	0.000103	0.001271
5595	Mitogen-activated protein kinase 3	MAPK3	0.625	3.48E−05	0.000581
3916	Lysosomal-associated membrane protein 1	LAMP1	0.608	6.33E−06	0.000174
81,631	MicrotubulE−associated protein 1 light chain 3 beta	MAP1LC3B	− 0.720	2.55E−06	8.96E−05
6767	Suppression of tumourigenicity 13 (colon carcinoma) (Hsp70 interacting protein)	ST13	− 0.954	2.3E−09	6.76E−07
65,018	PTEN induced putative kinase 1	PINK1	− 0.976	1.53E−05	0.00033
6794	Serine/threonine kinase 11	STK11	− 1.097	2.19E−10	1.11E−07
11,345	GABA(A) receptor-associated protein-like 2	GABARAPL2	− 1.108	2.6E−09	7.1E−07
2281	FK506 binding protein 1B, 12.6 kDa	FKBP1B	− 1.159	2.21E−05	0.000427
598	BCL2-like 1	BCL2L1	− 1.197	8.34E−07	4.12E−05
2309	Forkhead box O3	FOXO3	− 1.221	2.62E−07	1.82E−05
665	BCL2/adenovirus E1B 19 kDa interacting protein 3-like	BNIP3L	− 1.412	6.44E−08	6.77E−06

### GO and KEGG enrichment analysis of differentially expressed autophagy-related genes

**Fig. 3:**
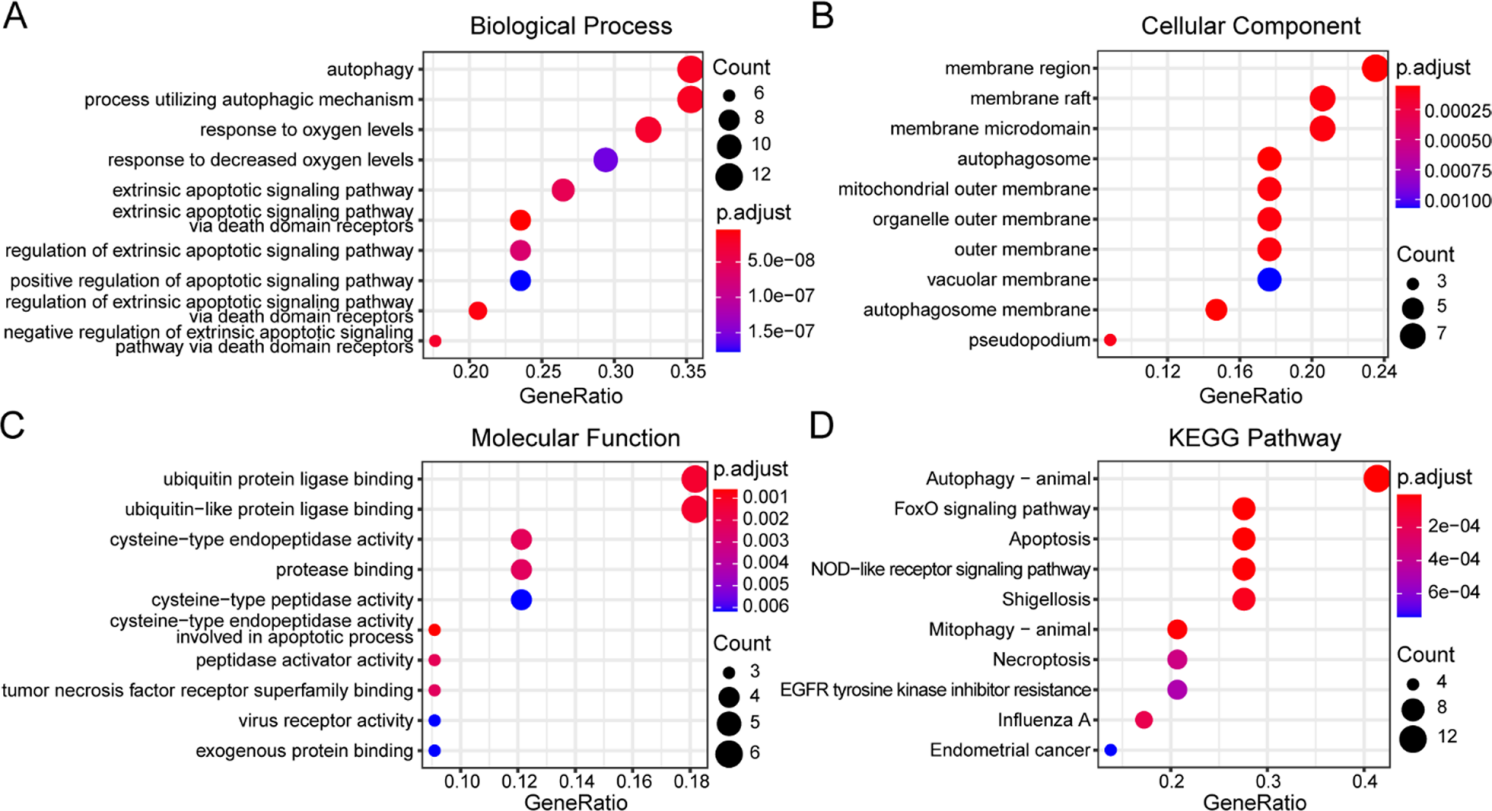
GO functional enrichment analysis and KEGG pathway analysis of 34 differentially expressed autophagy-related genes in SONFH and non-SONFH samples. **a** Bubble plot of the top 10 enriched BP terms of 34 targets. **b** Bubble plot of the top 10 enriched CC terms of 34 targets. **c** Bubble plot of top 10 enriched BP terms, CC terms and MF terms of 34 targets. **d** Bubble plot of top 10 enriched KEGG terms of 34 targets

**Fig. 4:**
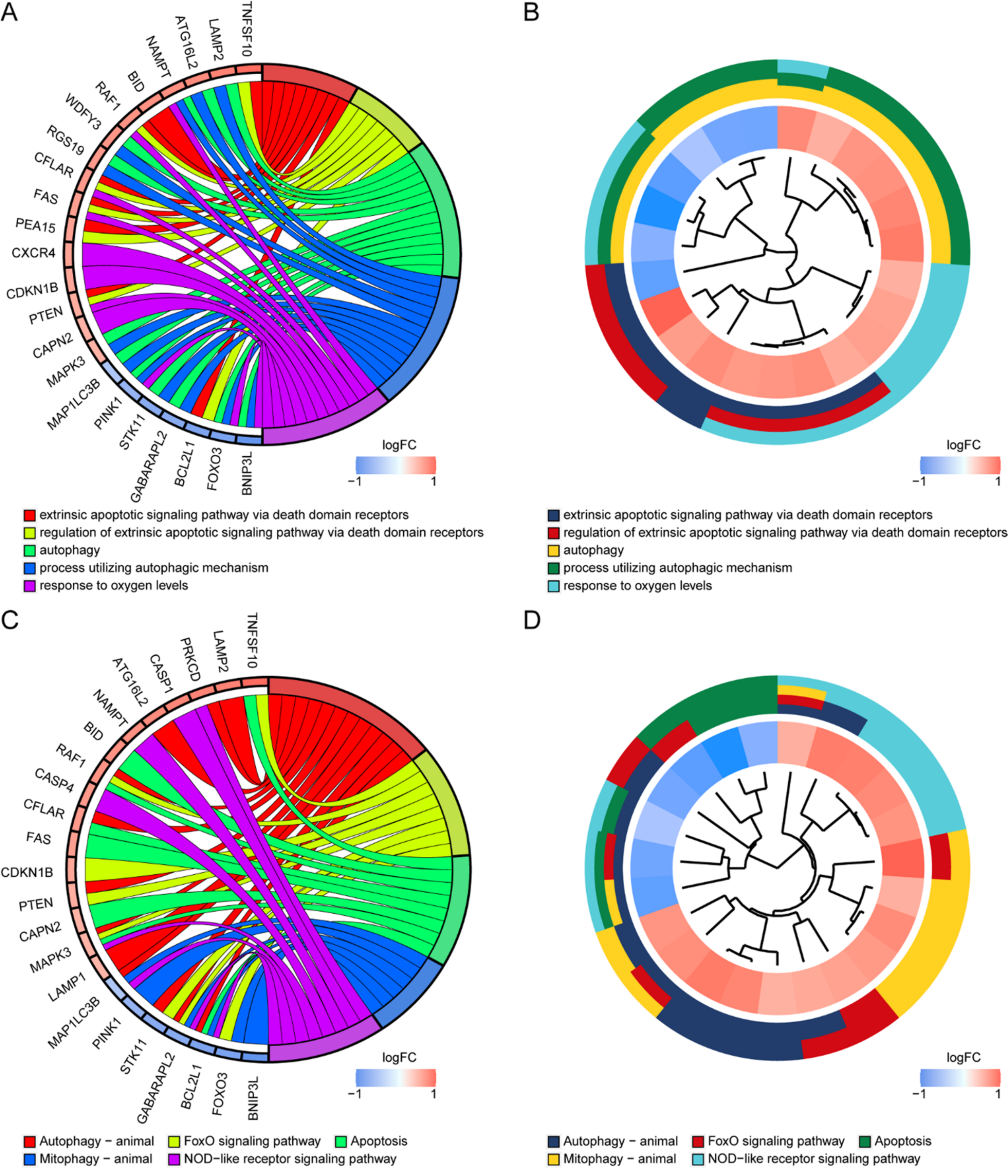
GO functional enrichment analysis and KEGG pathway analysis of 34 differentially expressed autophagy-related genes in SONFH and non-SONFH samples. **a** Relationship among the top 5 enriched BP terms and targets is represented in a chord plot. **b** Relationship among the top 5 enriched BP terms and targets is represented in a cluster plot. **c** Relationship among the top 5 enriched KEGG pathway terms and targets is represented in a chord plot. **d** The relationship between the top 5 enriched KEGG pathway terms and targets was represented by a cluster plot. The colours of the nodes range from red to blue in descending order of logFC values. The genes are ordered according to logFC values

To analyse the potential biological processes and KEGG pathways of these 34 differentially expressed autophagy-related genes, we conducted GO and KEGG enrichment analyses by using the “clusterProfiler” R package. There were 415 significantly enriched GO biological process terms, which were involved in the extrinsic apoptotic signalling pathway via death domain receptors, regulation of the extrinsic apoptotic signalling pathway via death domain receptors, autophagy, processes utilizing autophagic mechanisms and the response to oxygen levels, as shown in Fig. 3a, Fig. 4a, b. In the KEGG enrichment analysis, the 34 differentially expressed autophagy-related genes were significantly enriched in 69 KEGG pathway terms, such as autophagy, the FOXO signalling pathway, apoptosis, mitophagy and the NOD-like receptor signalling pathway, as shown in Fig. 3d, Fig. 4c, d.

### Correlation analysis of the differentially expressed autophagy-related genes

**Fig. 5:**
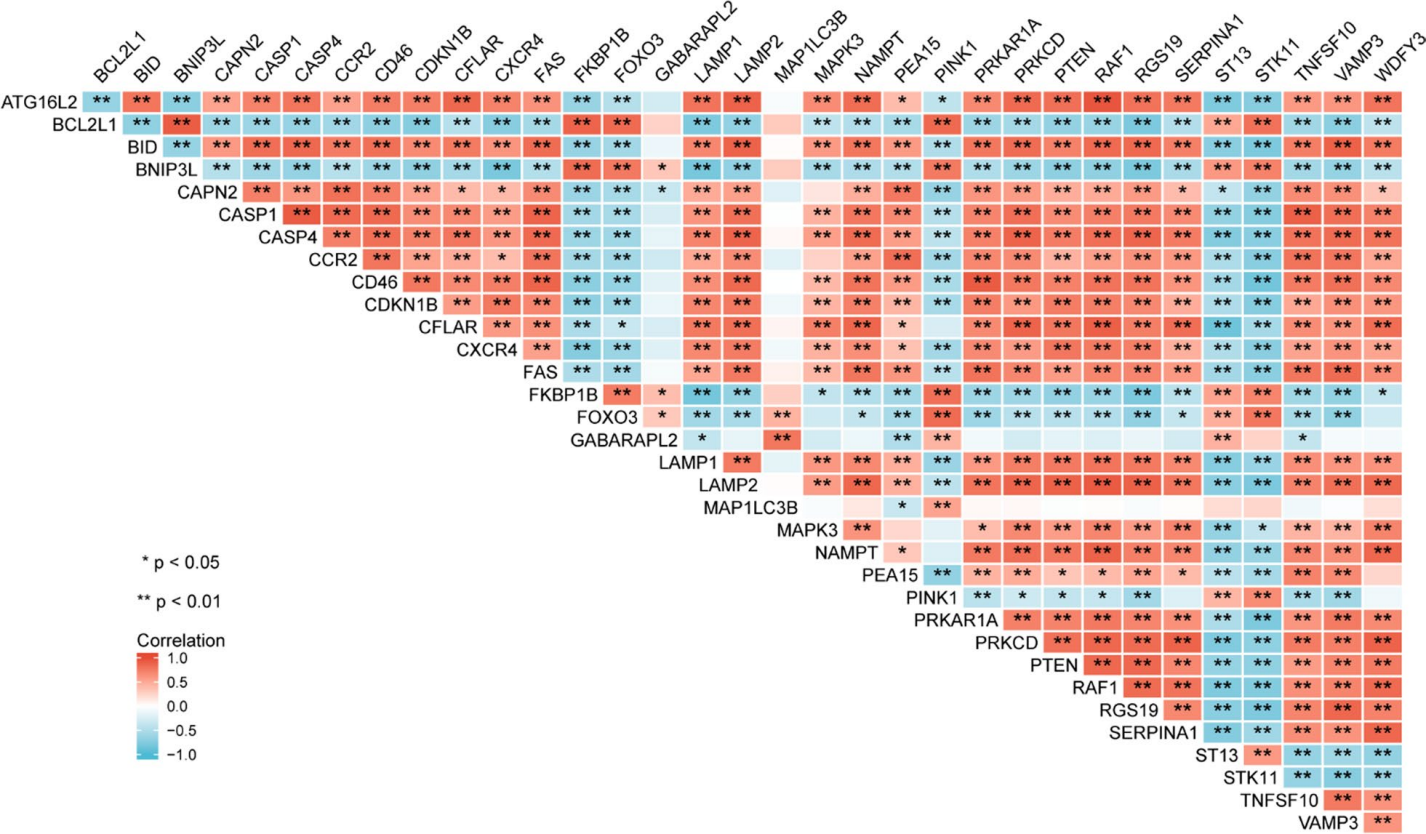
Spearman correlation analysis of the 34 differentially expressed autophagy-related genes in SONFH and non-SONFH samples

We performed correlation analysis to explore the expression correlation among these 34 autophagy-related genes in the GSE123568 dataset, and the results are shown in Fig. 5. The results suggested that some of these autophagy-related genes showed weak-to-moderate correlations. Among these 34 genes, RAF1 and ATG16L2 were most positively correlated (Cor = 0.95). Nevertheless, there were also some negatively correlated genes among these 34 genes, including ST13 and CFLAR (Cor =  − 0.86).

### PPI network and screening of key modules and hub genes

**Fig. 6:**
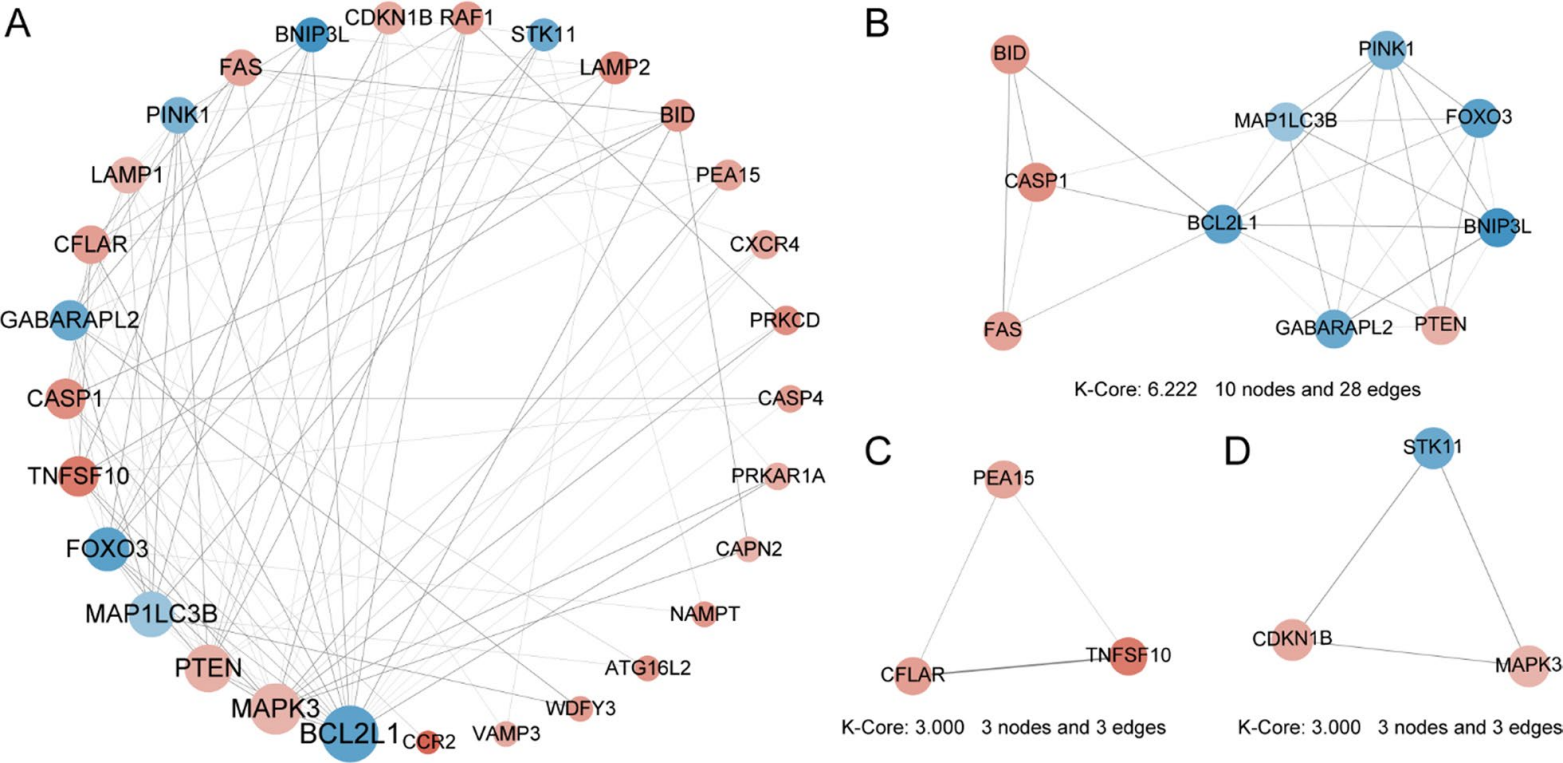
PPI network of 34 differentially expressed autophagy-related genes and three cluster modules identified by MCODE in SONFH and non-SONFH samples. **a** The PPI network of differentially expressed autophagy-related genes in SONFH and non-SONFH samples consists of 29 nodes and 102 edges. Each node represents a protein, while each edge represents one protein–protein interaction. The colour of the nodes reflects the fold change, where red indicates a more upregulated gene, and blue indicates a more downregulated gene; the size of the nodes reflects the degree value, where the larger the node, the greater the degree value. **b** Cluster 1: Score 6.222, 10 nodes and 28 edges; **c** Cluster 2: Score 3.000, 3 nodes and 3 edges; **d** Cluster 3: Score 3.000, 3 nodes and 3 edges

To determine the in-depth interactions among the 34 differentially expressed autophagy-related genes, PPI analysis was performed using the STRING database. These autophagy-related genes interacted with each other and the number of interactions of each gene was visualized with Cytoscape as shown in Fig. 6a. To identify the significant gene cluster modules, we used the MCODE plugin. The results revealed 3 clusters as shown in Fig. 6b–d. Cluster 1, composed of 10 nodes and 28 edges, comprising 4 upregulated and 6 downregulated differentially expressed genes, presented the highest cluster score (score: 6.222), followed by cluster 2 and cluster 3, which were both composed of 3 nodes and 3 edges (score: 3.000). To identify hub genes, the CytoHubba plugin was used. The results demonstrated that 10 hub genes could be identified by the MCC algorithms of CytoHubba, including TNFSF10, BCL2L1, PINK1, MAP1LC3B, BNIP3L, GABARAPL2, CFLAR, PTEN, FOXO3 and MAPK3, as shown in Table 3.

**Table 3:** 10 hub genes identified by the MCC algorithms of CytoHubba

Ensembl ID	Gene symbol	Degree	MCODE cluster	MCODE score
ENSP00000302564	BCL2L1	19	Cluster 1	6
ENSP00000370003	BNIP3L	7	Cluster 1	6
ENSP00000312455	CFLAR	9	Cluster 2	4.286
ENSP00000385824	FOXO3	12	Cluster 1	6
ENSP00000037243	GABARAPL2	10	Cluster 1	6
ENSP00000268607	MAP1LC3B	13	Cluster 1	6
ENSP00000263025	MAPK3	16	Cluster 3	3.364
ENSP00000364204	PINK1	8	Cluster 1	6
ENSP00000361021	PTEN	14	Cluster 1	6
ENSP00000241261	TNFSF10	10	Cluster 2	4.286

### Validation of differentially expressed autophagy-related genes in the GSE74089 dataset

**Fig. 7:**
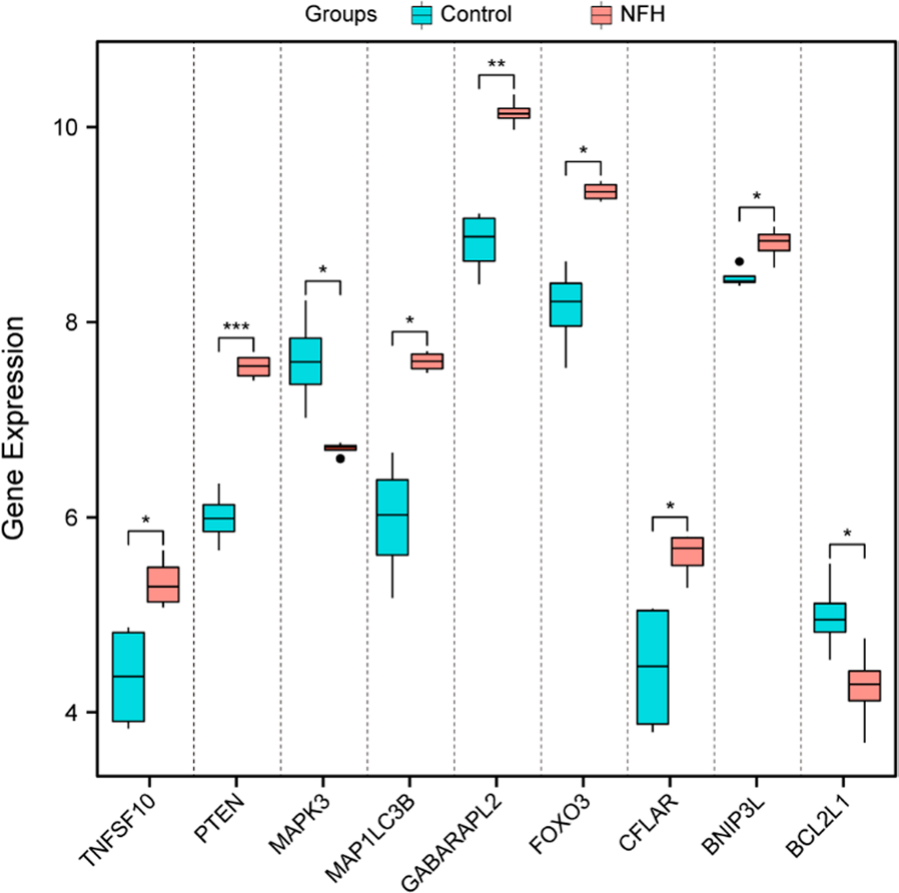
Boxplot of the 10 differentially expressed autophagy-related genes in NFH and healthy samples from the GSE74089 dataset

To verify the reliability of the GSE123568 dataset, the expression levels of 10 hub genes among the differentially expressed autophagy-related genes were further identified by using the GSE74089 dataset. The GSE74089 microarray dataset used for the identification of potential biomarkers in hip articular cartilage specimens from NFHs, which included 4 NFH patients and 4 healthy controls, was downloaded from the GEO database. Similar to the results of the mRNA microarray analysis of blood samples, the expression levels of TNFSF10, PTEN and CFLAR were significantly increased, and the expression levels of BCL2L1 were significantly decreased in the SONFH hip articular cartilage specimens relative to the normal samples, which might play an important role in autophagy after steroid administration, as shown in Fig. 7. However, the expression levels of MAPK3, MAP1LC3B, GABARAPL2, FOXO3, and BNIP3L showed significant differences between the two groups, and contrary to the expression data in the GSE123568 dataset, PINK1 showed no detectable expression in the GSE74089 dataset.

**Fig. 8:**
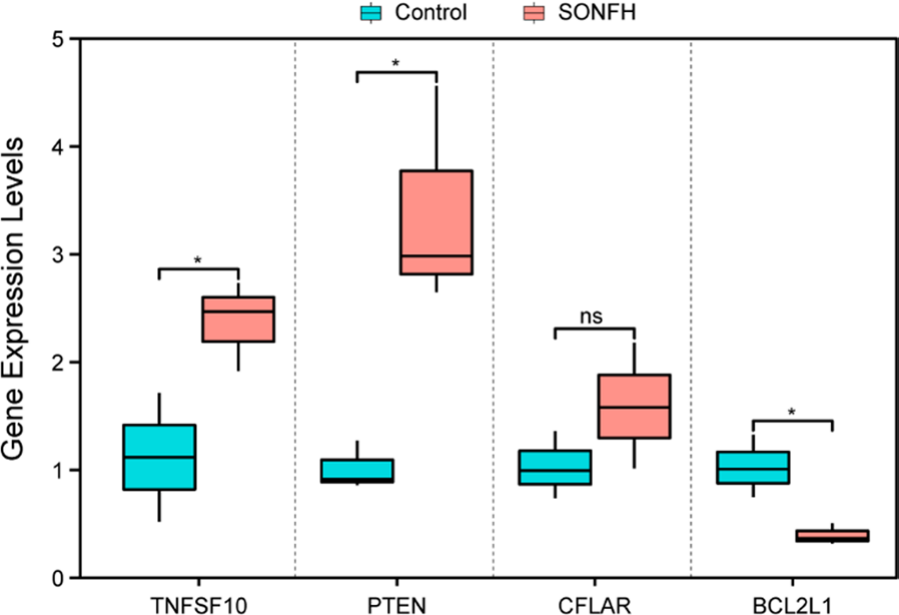
Validation of the expression of potential diagnostic markers including BCL2L1, TNFSF10, PTEN and CFLAR via qRT-PCR. *p < 0.05

The expression levels of BCL2L1, TNFSF10, PTEN and CFLAR in 12 cartilage samples were examined by qRT-PCR. Three diagnostic markers excluding CFLAR showed statistical significance (*p* < 0.05). TNFSF10 and PTEN were up-regulated in the SONFH samples compared with the healthy samples, while BCL2L1 was down-regulated in the SONFH samples compared with the healthy samples, but CFLAR was no statistically significant difference in the SONFH samples compared with the healthy samples, indicating that the above results are reproducible and reliable, as shown in Fig. 8.

## Discussion

SONFH, which is a common, progressive and refractory orthopaedic disease, is one of the most frequent causes of hip disability worldwide [16]. The main manifestation of SONFH in the early stage is a pathological change in bone trabeculae combined with a lack of adequate replacement of cells, and its clinical symptoms are not obvious. With the progression of SONFH, the patient's femoral head exhibits local sclerosis cystic changes, and even collapse, which seriously affects the normal life and work of the patient [17]. There are many joint preserving therapeutic approaches in the management of osteonecrosis of the femoral head; these treatments, such as core decompression (CD), CD combined with bone marrow-derived cell therapy and osteotomies, can prevent or delay the necessity of total hip arthroplasty (THA) [18–20]. The exact pathogenesis of SONFH has not been defined, but with the accumulation of studies on femoral head necrosis, many hypotheses about SONFH pathogenicity have been proposed to explain the underlying mechanisms, such as programmed cell death [21–23]. Programmed cell death is an active process controlled by the related genes that regulates the development of the body and maintains a stable internal environment in multicellular organisms [24]. Autophagy is the key common form of programmed cell death in organisms, and regulates the negative effects of GC on osteoblasts, osteoclasts and osteocytes [25]. However, the potential biomarkers and regulatory mechanisms of autophagy involved in the pathological process of SONFH remain unclear. Therefore, the present study explored several genes that have not previously been mentioned in the context of autophagy and SONFH, which would provide an effective reference and important guidance for the clinical diagnosis and treatment of SONFH patients.

To the best of our knowledge, only a few published articles related to bone metabolic diseases have explored autophagy-related genes. A review of the role of autophagy in bone homeostasis and the onset of osteoporosis introduced the relevance of autophagy to bone physiology and discussed its role and therapeutic potential in the pathogenesis of osteoporosis [26]. However, the bioinformatics analysis of autophagy-related genes has not previously been explored in SONFH. In this study, we identified the first 34 potential differentially expressed autophagy-related genes associated with SONFH based on the intersection of differentially expressed genes from the GSE123568 dataset and the autophagy-related HADb via bioinformatics analysis; these genes included 25 upregulated genes and 9 downregulated genes. Some of these differentially expressed autophagy-related genes involved in SONFH have been previously studied. For example, Ye et al. demonstrated that phosphatase and tensin homologue (PTEN), a tumour suppressor gene that promotes cell apoptosis, was elevated in SONFH patients [27]. Chen et al. discovered eight promising serum biomarkers, including PTEN, as a potentially new non-invasive diagnostic tool for the detection of SONFH, which was similar to the results of Wang et al. In addition, accumulating evidence suggests that FAS mRNA expression levels in MC3T3-E1 cells are correlated with bone or osteoblast cell apoptosis in SONFH patients [28]. Additional studies will be needed to confirm the potential differentially expressed autophagy-related genes in SONFH.

To clarify the underlying molecular mechanisms of the 34 differentially expressed autophagy-related genes involved in SONFH, we evaluated their biological functions through GO and KEGG enrichment analysis. The GO and KEGG enrichment analyses revealed that the differentially expressed autophagy-related genes involved in SONFH were significantly enriched in several terms related to autophagy, apoptosis, mitophagy, the FOXO signalling pathway, and the NOD-like receptor signalling pathway. Previous studies of the FOXO signalling pathway have focused on the regulation of bone cell homeostasis via the FOXO induction effect on autophagy [29]. A previous bioinformatics-based study demonstrated that the Toll-like receptor (TLR), neurotrophin and NOD-like receptor signalling pathways were most likely to be regulated by the differential miRNAs involved in SONFH [30]. Another recent study showed that GC could induce the autophagy and apoptosis of bone cells, which was related to the dose of GC (i.e. apoptosis was activated by a high dose of GC, while a low dose of GC causes autophagy) [31]. Clearly, many more studies will be required to corroborate our results and produce more insights into the potential biological functions of these differentially expressed autophagy-related genes in SONFH.

Thereafter, the PPI network of differentially expressed autophagy-related genes involved in SONFH was constructed by using the STRING database, and the node topological characteristics and internode interactions were analysed to reveal the biological mechanisms related to the PPI network with the NetworkAnalyzer plugin of Cytoscape. Biological networks might consist of several functional modules, whose complex subunits and their interactions often lead to the same biological processes, providing a new perspective into the biological functions that constitute various components of the complex network. A total of 3 cluster modules from the PPI network were extracted by MCODE analysis. Cluster 1, which comprised 10 nodes and 28 edges, showed the highest cluster score; this cluster included 4 upregulated and 6 downregulated differentially expressed genes including PINK1, BCL2L1, BNIP3L, MAP1LC3B, GABARAPL2, CASP1, PTEN, FAS, BID and FOXO3. The filtered hub nodes also varied according to different filtering criteria. The top 10 hub genes involved in autophagy were further identified by the MCC algorithms of CytoHubba with reference to Gov E; these genes included TNFSF10, BCL2L1, PINK1, MAP1LC3B, BNIP3L, GABARAPL2, CFLAR, PTEN, FOXO3, and MAPK3 [32]. All 10 hub genes identified from CytoHubba were present within the intersection of the CytoHubba and 3 cluster module results.

Based on the bioinformatics analysis results of the GSE123568 dataset from blood samples, the expression levels of the top 10 differentially expressed autophagy-related hub genes were further identified from the GSE74089 dataset of hip articular cartilage specimens. TNFSF10, PTEN and CFLAR were significantly upregulated, while BCL2L1 was significantly downregulated, which was consistent between the GSE123568 and GSE123568 datasets. TNFSF10, PTEN and BCL2L1 further validated by the PCR, with a consistent expression. Several studies on these consistently expressed genes have been reported them to be associated with bone metabolic diseases or autophagy. For instance, Komori et al. indicated that BCL2L1 overexpression in osteoblasts increased the volume of trabecular and cortical bone with normal structures and maintained it primarily by preventing osteoblast apoptosis in osteoporosis [33]. Liu and colleagues proposed that TNFSF10 overexpression probably stimulated proliferation and inflammation and inhibited apoptosis by regulating the miR-376-3p/FGFR1 axis in osteoarthritis [34]. Xu et al. demonstrated that PTEN affects the risk of osteoarthritis development by regulating the expression of autophagy regulators and the mTOR pathway [35]. Wang et al. found VO-OHpic (a potent inhibitor of PTEN) could prevent and treat SONFH by attenuating the pathological processes including impaired angiogenesis, abnormal apoptosis, thrombosis and fat embolism caused by the dysfunction of bone endothelial cells [36]. However, few related studies on consistently expressed genes other than PTEN have been published in the context of autophagy and SONFH. These novel genes provide new concepts related to autophagy after SONFH, and further investigations will be aimed at better understanding this topic.

Although potential autophagy-related genes involved in SONFH were identified based on bioinformatics, there were still some limitations of this study. First, although the GEO database stores large amounts of high-throughput data, we did not find additional underutilized datasets of SONFH, resulting in a particularly limited number of samples in this study. Second, further in vitro and in vivo experiments to validate the differentially expressed autophagy-related genes and their potential mechanisms are lacking. Therefore, further research is warranted to address the possible limitations in terms of biased results and conclusions.

## Conclusion

In conclusion, we identified 34 potential autophagy-related genes involved in SONFH with the help of a bioinformatic analysis toolkit in the present study. Moreover, the consistently expressed hub genes TNFSF10, PTEN and BCL2L1 might serve as potential therapeutic targets and biomarkers of autophagy after SONFH. These results will facilitate the in-depth exploration of the development of SONFH by regulating autophagy and provide novel insights into the diagnosis and prognosis of SONFH.

## Data Availability

Publicly available datasets were analysed in this study. These datasets can be found at the following URLs: HADb (http://www.autophagy.lu/index.html), GSE123568 (https://www.ncbi.nlm.nih.gov/geo/query/acc.cgi?acc=GSE123568) and GSE74089 (https://www.ncbi.nlm.nih.gov/geo/query/acc.cgi?acc=GSE74089).

## Abbreviations

BP: Biological process
BN: Bottleneck
CC: Cellular component
DMNC: Density of Maximum Neighbourhood Component
EPC: Edge Percolated Component
FC: Fold change
GC: Glucocorticoids
GEO: Gene Expression Omnibus
GO: Gene Ontology
HADb: Human Autophagy Database
KEGG: Kyoto Encyclopedia of Genes and Genomes
MCODE: Molecular complex detection
MCC: Maximal Clique Centrality
MF: Molecular function
MNC: Maximum Neighbourhood Component
NCBI: National Center for Biotechnology Information
NFH: Necrotic femur head
PCA: Principal component analysis
PPI: Protein–protein interaction
PTEN: Phosphatase and tensin homologue
RMA: Robust multiarray average
SONFH: Steroid-induced osteonecrosis of the femoral head
STRING: Search Tool for Recurring Instances of Neighbouring Genes
TLR: Toll-like receptor
